# Personal air sampling for pesticides in the California San Joaquin Valley

**DOI:** 10.1038/s41370-024-00708-4

**Published:** 2024-09-10

**Authors:** Deborah H. Bennett, Jane Sellen, Rebecca Moran, Christopher P. Alaimo, Thomas M. Young

**Affiliations:** 1 Department of Public Health Sciences, University of California at Davis, Davis, CA USA; 2 Californians for Pesticide Reform, Berkeley, CA USA; 3 Department of Civil and Environmental Engineering, University of California at Davis, Davis, CA USA

**Keywords:** Pesticides, San Joaquin Valley, Exposure, Agriculture

## Abstract

**Background:**

California is a leading agricultural state and with that, has significant applications of pesticides. Levels of exposure have been measured to be higher among residents in agricultural areas, but measures of personal inhalation exposure to a wide range of pesticides are lacking. Community members in the San Joaquin Valley have expressed concern over pesticide exposures. Working with community members, a wide range of pesticides in personal air samples were measured.

**Methods:**

Adult and school-aged participants were recruited from small agricultural towns in the San Joaquin Valley. Participants wore a backpack sampler for 8–14 h on 1–3 days. Samples were collected on two tubes, one with Tenax-TA resin and the other with XAD-2 resin. In total, 21 pesticides were analyzed using both LC/MS and GC/MS methods.

**Results:**

Thirty-one adult participants and 11 school aged participants were recruited, and sampling occurred on a total of 92 days. Seven adults, 22% of adult participants, and one school child had detectable levels of at least one pesticide. Pesticides detected above the limit of detection were 1,3-dichloropropene, chlorpyrifos, pyrimethanil, burprofezin and penthiopyrad. When these samples were collected, chlorpyrifos was not permitted to be used in California.

**Impact statement:**

- California, a leading agricultural state, has significant pesticide use, leading to concern about exposures among community members. Thirty-one adult and 11 school aged participants wore personal air sampling backpacks from 1–3 days. Twenty-two percent of adult participants had detectable levels of at least one pesticide. Two pesticides with established toxicity, 1,3-dichloropropene and chlorpyrifos were detected, along with first time measurements of pyrimethanil, burprofezin and penthiopyrad in the United States, which all have potential indications of toxicity. This study suggests the need to expand which pesticides are measured in agricultural communities.

## Introduction

California is a leading agricultural state and as such, uses a significant amount of pesticides, with 191 million pounds of pesticide active ingredients applied statewide in 2021 [1]. Pesticides are by design toxic to target organisms and have been found to be associated with a number of adverse human health effects [2–6]. When pesticides are sprayed on fields, there is both primary drift on the day of the application as well as secondary drift in the days that follow resulting from volatilization and processes such as wind erosion, movement of soil particles and particle-associated pesticide drift from wind [7, 8].

Levels of exposure have been found to be higher for residents living near agricultural fields, specifically higher levels of pesticide metabolites in urine [9–11] and of pesticides in dust in their homes [12–18]. Members of agricultural communities are often interested in their personal exposure to pesticides. For example, a community group recently motivated a study utilizing silicone bracelets worn by Latina girls (14–16 years old) to determine if their exposures were higher than in comparison groups. The study found higher concentrations in wristbands worn by individuals with the greatest proximity to nearby agricultural use. Levels were also influenced by individual household characteristics, such as carpeting and not having a doormat at the house [19]. Biomarkers of exposure using urine are only available for a small number of pesticides, with new methods quantifying levels in serum [20] and hair [21] only just beginning to be used for a broader range of pesticides. Levels in dust can be used as a marker of exposure, but cannot currently be used to directly quantify exposure. Numerous studies conducted in California have found increased adverse health impacts for children whose mothers lived near pesticide applications, including an increase in the rate of developing childhood acute lymphoblastic and acute myeloid leukemia [22], autism [23, 24], neural tube defects [25], and having lower birthweight [26] and lower Full-Scale Intelligence Quotient [27, 28].

The largest agricultural region in California is the San Joaquin Valley. The top five counties in California in terms of mass of pesticide active ingredient applied are Fresno, Kern, Tulare, San Joaquin, and Madera Counties, all located in the San Joaquin Valley [1]. A recent review considering biological and environmental samples from 27 studies across the globe found that determinants for increased exposure included proximity to fields, crop acreage around residence, and amount of pesticide applied around the residence [29]. Residents living in this region have long expressed concerns about potential impacts of pesticide exposure on their health [30, 31]. The San Joaquin Valley not only has high levels of pesticides applied, but also air and water pollution [32, 33]. In addition, the San Joaquin Valley has high rates of poverty, with just over 20% of the population having incomes in 2020 under the federal poverty line for a family of 4 [34]. One of the major metropolitan areas in the region, Fresno, had the second highest concentrated poverty rate in the country in 2018, based on the number of families living in neighborhoods with a poverty rate exceeding 40% [35].

This current study was motivated by the concern of residents in the San Joaquin Valley of California regarding their exposure to pesticides in their communities. The goal of this study is to quantify individual pesticide exposures among adults and school-aged children in this agricultural region. In addition, this study is among the first to be conducted after the initiation of two recent regulations in California. The first regulation restricted pesticide applications from 6:00 AM to 6:00 PM Monday through Friday within 0.25 miles of public schools and daycare centers that became effective on January 1, 2018 [36]. The second regulation banned all sales of chlorpyrifos after February 6, 2020, with no use of existing stockpiles permitted after December 31, 2020 with the exception of a limited number of granular products containing chlorpyrifos [37].

## Methods

### Recruitment

Participants were recruited to wear sampling backpacks while they went about their daily activities. Each volunteer participated in the study for between 1 and 3 days. There were two groups, adults and high-school children. Participants were recruited from 2–3 small agricultural towns in each of Fresno, Tulare, and Kern Counties, the counties with the highest pesticide applications by mass of active ingredient in CA [1]. Because some of the towns selected were very small (2 with populations <500), we are not specifying names of the towns to protect the identities of participants. Community groups and local non-profit organizations assisted in recruiting participants for this study. They held community meetings, distributed fliers at community events, and gave fliers to individual households. Adults who were interested in participating contacted researchers by phone or e-mail. School children were recruited through meetings at schools in eligible counties. Study protocols were approved by the Institutional Review Board at the University of California, Davis, and written informed consent was obtained from all participants.

### Pesticide selection

Pesticides were selected for inclusion on the target analyte list using several approaches, with all pesticides included listed in Table 1. First, the community partners were interested in 1,3-dichloropropene and the class of organophosphate pesticides, and the university researchers were interested in the class of pyrethroid pesticides, all based on the known adverse health impacts of these pesticides and thus a number of these pesticides were selected. Second, we selected pesticides to which participants in the study area have high exposure risk based on nearby applications. The California Department of Pesticide Regulation requires applicators to report use of all pesticides to the state through the California Pesticide-Use Report. We used this database to determine which pesticides were heavily used near each specific town in the two most recent years in the spring when we planned to sample. We additionally considered the vapor pressure in the selection process to increase probability of detection in the air samples. Priority was also given to pesticides that had indication of potential toxicity based on several high-throughput tests, specifically those utilized in a recent effort to prioritize chemicals for inclusion in a nationwide childhood health program [38], focusing on neurological, endocrine, and obesity processes. For neurological endpoints, we included 3 calcium, 1 ligand, 3 potassium, and 1 sodium ion-channel assay, all in the ToxCast™ database [39] as well as an integrated assay that measures neural network activity [40]. The 4 main endocrine processes evaluated were estrogen, androgen, thyroid, and steroidogenic using scientific papers that merged results from several assays, producing an overall score [41–43], as well as additional thyroid related assays [44, 45]. Several biological processes have been associated with diabetes and obesity (insulin sensitivity in peripheral tissue, pancreatic islet and β-cell function, adipocyte differentiation, and feeding behavior), and high throughput assay results relevant to these processes were used [46]. Finally, the pesticides needed to be able to be analyzed on one of the planned sampling platforms (gas chromatography electron ionization mass spectrometry and liquid chromatography electrospray ionization mass spectrometry).

**Table 1:** Pesticides included for analysis in this study.

Pesticide	Tulare (lbs applied)	Kern (lbs applied)	Fresno (lbs applied)	Analytical Method	Detected in Study	Vapor Pressure (Pa)	Number of High-Throughput Assay Hits	Pot-entially Neuro- toxic
**Halogenated Hydrocarbon Fumigant**								
1,3-Dichloropropene	781,284	1,573,440	1,376,352	TD-GC/MS	X	3.11E + 01	NA	
**Pyrethroid Insecticides**								
Bifenthrin	16,792	40,978	58,024	GC/MS - NCI		1.79E-07	0	
lambda - Cyhalothrin	5242	8377	15,474	GC/MS - NCI		7.50E-09	NA	
Permethrin	5969	7074	13,586	GC/MS - NCI		2.19E-08	1	
**Organophosphate Insecticides**								
Chlorpyrifos	249	2295	468	GC/MS - NCI	X	2.03E-05	2	
Naled	2062	6461	23,364	GC/MS - EI		2.01E-04	0	
Dimethoate	9683	6538	30,045	GC/MS - EI		1.87E-05	2	
**Carbamate Insecticides**								
Formetanate Hydrochloride	20,202	9154	13,007	LC/MS		1.22E-08	1	
**Other Insecticides**								
Buprofezin	59,777	72,006	34,292	GC/MS - EI	X	9.36E-06	1	X
Thiamethoxam	7927	2218	5582	LC/MS		1.54E-06	1	X
Pyriproxyfen	5426	4492	3972	LC/MS		2.17E-06	1	X
**Fungicides**								
Pyrimethanil	3933	11,798	16,308	GC/MS - EI	X	1.64E-05	1	
Tebuconazole	6811	9485	13,510	GC/MS - EI		1.29E-08	6	X
Fenhexamid	6353	17,935	2315	LC/MS		3.44E-08	4	
Triflumizole	2699	5833	3115	LC/MS		1.40E-06	8	X
Trifloxystrobin	10,279	13,919	18,267	LC/MS		2.70E-08	2	X
Penthiopyrad	10,683	15,576	32,590	LC/MS	X	1.26E-06	NA	
**Herbicides**								
Oxyfluorfen	57,528	134,693	142,699	LC/MS		2.01E-07	1	X
Linuron	–	16,739	2055	LC/MS		1.45E-06	3	X
Trifluralin	5635	5920	40,731	GC/MS - EI		4.60E-05	1	X
(S)-Metolachlor	3597	13,635	53,919	LC/MS		3.11E-05	NA	

### Sample collection

On any given sampling day, participants were asked to wear a personal sampler, which was integrated into a small backpack, with two sampling tubes attached to the backpack strap at shoulder level in the personal breathing zone with a Leland Legacy personal sampling pump (SKC Inc., Eighty Four, PA) with a noise-dampening cover housed in the main backpack compartment [47]. Participants wore the sampler for approximately 8–14 h, turning the pumps on shortly after getting up in the morning and turning them off at the end of the day, or end of the school day for school age children.

Participants met with researchers before they started sampling to receive the personal sampler, learn how to turn the pump on and off, and to learn to change the sampling tubes between sample days, if needed. They were instructed to keep the tubes refrigerated at the end of the sampling day. Participants were also asked to fill out a brief activity log noting the activities they conducted that day.

Typically, there were two sample tubes, a thermal desorption tube packed with Tenax-TA resin (Part No. C1-AAXX-5003, Markes International) and an OVS tube packed with XAD-2 resin and a glass fiber filter for trapping aerosols (Part No. 226-58, SKC, Inc). In the first year, the target flow rate of the Tenax tube was set at 30 ml/min, such that the target sample volume was between 15 and 25 L, and the target flow rate of the OVS tube was 300 ml/min, with a target sample volume of between 150 and 250 L. In the second year, we wanted to increase the sample volume on the OVS tube, while keeping the same approximate volume on the Tenax tube and still maintaining a system that operated on one pump, which limits the range of flow to each tube. Some adult participants used Tenax at 15 ml/min and OVS at 400 ml/min, wearing the sampling tubes for 2 days of sampling instead of just 1 day of sampling while the school children and some of the other adult participants used Tenax at 50 ml/min and OVS at 450 ml/min for a single day.

### Sample analysis

Tenax-TA thermal desorption tubes were analyzed on a Unity 2 thermal desorption system (Markes International) interfaced with a gas chromatograph and mass spectrometer (GC/MS). Thermal desorption tubes require no pre-treatment prior to analysis. Sample tubes were thermally desorbed using the Unity 2 thermal desorption unit, which under a flow of helium (10 ml/min) heats the tube to 250 °C for 5 min, recollecting the sample onto a TO-17-specific cold trap. This trap was then rapidly heated to 280 °C, with the flow from the trap being transferred directly onto the analytical GC column. Sample analysis was carried out on an Agilent 6890 N gas chromatograph coupled with an Agilent 5973 N single quadrupole mass spectrometer. Separation was achieved using an Agilent DB-VRX column (60 m x 0.25 mm, 1.4 μm film thickness) using helium as the carrier gas. The GC oven was held at 35 °C for 5 min, before ramping up to 250 °C at 7.5 °C/min, with a final hold time of 10 min. The sole analyte, 1,3-dichloropropene, was quantified by comparison to a 7-point calibration curve prepared using a fixed-concentration gaseous standard drawn through a series of thermal desorption tubes for varying lengths of time [48]. The calibration curve had an R^2^ value of 0.9999 and an LOQ of 0.27 ng on-column.

OVS sorbent tubes, along with the glass fiber filter, were extracted by transferring each section of the sorbent (front and back) into labeled glass vials, to which 2 ml of a 1:1 hexane acetone solution was added. The vials were then capped and sonicated for 30 min, and the supernatant liquid transferred to evaporation tubes and concentrated to 1 ml under a stream of nitrogen. The extract was then split into two equal halves, one for GC analysis and the other for LC analysis. The GC sample was further concentrated to 200 ul and was spiked with 10 ul of an internal standard solution. The LC sample was evaporated to near dryness and reconstituted in 1 ml of methanol. All OVS sorbent extract analytes were quantified by comparison to a calibration curve prepared using a mixture of analyte standards.

The following pesticides were analyzed via GC/MS negative chemical ionization (NCI) on the OVS tube: Chlorpyrifos, Bifenthrin, Cyhalothrin, and Permethrin [49]. Sample analysis was carried out on an Agilent 6890 N gas chromatograph coupled with an Agilent 5973 N single quadrupole mass spectrometer configured for chemical ionization. Separation was achieved using an Agilent DB-5 column (30 m × 0.25 mm, 0.25 μm film thickness) using helium as the carrier gas. The GC oven was held at 100 °C for 1 min, before ramping up to 240 °C at 25 °C/min, followed by ramping to 280 °C at 3.8 °C/min, with a final hold time of 5 min. Chlorpyrifos was quantified by comparison to an 8-point calibration curve with an R^2^ value of 0.9989 and an LOQ of 5 pg on-column.

The following pesticides were analyzed via GC/MS electron ionization (EI) on the OVS tube: Naled, Trifluralin, Dimethoate, Pyrimethanil, Burprofezin, and Tebuconazole [50, 51]. Sample analysis was carried out on an Agilent 8890 gas chromatograph coupled with an Agilent 7250 quadrupole/time-of-flight mass spectrometer. Separation was achieved using an Agilent DB-5 column (30 m x 0.25 mm, 0.25 μm film thickness) using helium as the carrier gas. The GC oven was held at 35 °C for 3 min, before ramping up to 325 °C at 8 °C/min, with a final hold time of 3 min. Buprofezin and Pyrimethanil were quantified by comparison to a 6-point calibration curve with an R^2^ value of over 0.99 and an LOQ of 5 pg on-column.

The following pesticides were analyzed via LC/MS on the OVS tube: Formetanate, Thiamethoxam, Linuron, Fenhexamid, Triflumizole, S-Metolachlor, Trifloxystrobin, Penthiopyrad, Oxyfluorfen, and Pyriproxyfen. Sample analysis was carried out on an Agilent 6530 LC-qTOF-MS. Separation was achieved using an Agilent Zorbax Eclipse Plus C18 column (2.1 x 100 mm, 1.8 um particle size) using water and acetonitrile as the mobile phase solvents. The solvent gradient was held at 2% organic for 1.5 min before ramping up to 100% organic at 16.5 min, where it was held for an additional 4 min. Penthiopyrad was quantified by comparison to a 12-point calibration curve with an R^2^ value of 0.9986 with an LOQ of 1 pg on-column.

## Results

### Study participants

A total of 31 adult participants were recruited in 2021, each wearing a sampling backpack for either 1 or 2 days, for a total of 42 sampling days, with a single Tenax and a single OVS tube collected on each day. Sampling occurred from May to July 2021, which was towards the later part of the pesticide application period in the region. In 2022, 14 of the 31 adults who participated in 2021 participated again, wearing the backpack 1 to 2 days, for a total of 21 of each type of sampling tubes. In addition, 11 school-aged children participated, none of whom were related to the adult participants. Each wore the backpack 2 to 3 days, for a total of 27 Tenax tubes and 29 OVS tubes collected. Sampling occurred from February to April 2022, with the earlier time-period selected to capture the earlier part of the pesticide application period. A total of 90 Tenax tubes and 92 OVS tubes were collected in the study.

We note that while the intent of this study was not to focus particularly on agricultural work, 7 participants indicated on their activity log that they participated in agriculture work, with 2 more reporting work in packing houses, on the day they wore the samplers. None of the participants reported applying pesticides on the days they worked.

### Measured concentrations

Seven adults and one school child had detectable levels of at least one pesticide, with two participants having two pesticides detected in their sample, and one participant having detectable levels on two consecutive days (1,3-dichloropropene). Overall, 22% of the adult participants (7 out of 31) had at least one detectable concentration during the time they participated in the study. Specifically, the following compounds and concentrations were measured.

- Chlorpyrifos was detected twice in Tulare County, once in 2021 (an adult) and once in 2022 (a child) with concentrations of 28 and 13 ng/m^3^.
- Pyrimethanil was detected once in Kern County in 2021 with concentrations of 12 ng/m^3^.
- Burprofezin was detected three times, all in 2021, in both Fresno and Kern Counties with concentrations of 20, 28, and 25 ng/m^3^.
- Penthiopyrad was detected once in 2021 in Fresno County with concentrations of 1.2 ng/m^3^.
- 1,3-dichloropropene was detected three times in 2021 in Fresno County with concentrations of 90, 140, and 60 ng/m^3^. One participant had detectable levels on two consecutive days and the other participant additionally had exposure to burprofezin.
- Trifluralin was detected once in Kern County in 2021, but the concentration was below the limit of quantitation (LOQ), and was detected in the same sample as the sample noted above with pyrimethanil detected.

The adult participant who had a sample with a detectable level of chlorpyrifos reported that they participated in agriculture work that day. The child participant went to the same school that day as other participants, and those participants did not have detectable levels, indicating that the source of chlorpyrifos exposure was not necessarily the school.

Of the seven participants reporting they participated in agricultural work, three had detectable levels of pesticide. One was exposed to chlorpyrifos (noted above), one was exposed to pyrimethanil and trifluralin, and the third to burprofezin.

The participants were each given a report indicating if they had been exposed to a pesticide on any of their sampling days, and if they were, information about that pesticide. They were additionally given a summary of the findings of the report. A community meeting was held to present the results of the study to the broader community.

## Discussion

### Points of comparison for exposure

The California Department of Pesticide Regulation (CDPR) began conducting air monitoring in 2006. In that year, samples were collected for 3 days a week for the entire year in a farming community in the San Joaquin Valley, measuring quantifiable levels of chlorpyrifos in 64% of the samples with an average concentration of 23 ng/m^3^ [52]. Since then, frequency of detection and levels have decreased. For chlorpyrifos, between 2018 and 2022, detectable levels were measured in 9.3% of samples in 2018, decreasing to 1.2% in 2019, and then no detections in 2020 and 2021, with 0.45% detectable (below quantification) in 2022 [53–57]. The highest measured concentration in the San Juaquin Valley was 50 ng/m^3^. Chlorpyrifos was measured outdoors on 20 days in Tulare County in 1996, prior to application of use restrictions, and the median 24-h concentration was 33 ng/m^3^ [58]. A study in New Zealand measured concentrations both at a field where an application occurred and 0.5 km downwind of the field, measuring a concentration of 90 ng/m^3^ on the field the day of the application and 1 ng/m^3^ 2 days after the spraying 0.5 km downwind of the field [59]. Passive samplers were used to measure both indoor and outdoor levels of chlorpyrifos in agricultural regions of Washington State in 2011 [60]. Outdoor concentrations ranged from 9 to 199 ng/m^3^ while indoor levels ranged from <LOD −18 ng/m^3^. It is surprising that we measured chlorpyrifos at a level similar to these levels in one sample given that chlorpyrifos use was not permitted except in granular form when the sample was collected. Granular applications were noted by the California Department of Pesticide Regulations as be anticipated to have lower resulting air concentrations than other application methods and thus allowed for this use [61]. As noted, the participant was working in an agricultural field the day they wore the sampling backpack. For the school-aged participant, it is unknown where the exposure occurred.

The most commonly detected compound in the CA monitoring network is 1,3-dichloropropene, with quantifiable levels measured in, on average, 25% of samples from 2018 to 2022, ranging from 2.9–47% by year [53–57]. In 2022 in Shafter, the closest site to our sampling region, the highest 24-h concentration was 5242 ng/m^3^ and the average was 253 ng/m^3^ [57]. A study was conducted to measure 1,3-dichloropropene in Merced County in 2010–2011 and found an average concentration of 2500 ng/m^3^, driven by a maximum sample with a concentration of 369,000 ng/m^3^. Most of the samples collected in that study were below the LOQ of 0.05 μg/m^3^ [62]. A year-long measurement campaign conducted by CARB in conjunction with the CDPR detected 1,3-D in 34% of samples with an average concentration of 1970 ng/m^3^ [52]. The personal exposures measured in this study ranged from 60 to 140 ng/m^3^. However, the distributions of concentrations in prior studies had considerable variability, and many sampling days in previous studies had concentrations similar to those measured in this study.

Pyrimethanil was measured in Costa Rica at 12 schools near banana plantations in 2010 and 2011, with passive samplers, each set out on average for 6.7 weeks. The 10th percentile of exposure was 0.27 ng/m^3^, the median was 1.3 ng/m^3^, and the 90th percentile was 17 ng/m^3^ [63]. Pyrimethanil was also measured in Central Chile using passive samplers set out for 30, 60, or 90 days, with a maximum concentration of 0.053 ng/m^3^ [64]. Samples were also taken directly surrounding a field in New Zealand where the compound was being sprayed and concentrations ranged from 400 ng/m^3^ to 3200 ng/m^3^ [65]. This study measured 12 ng/m^3^, similar to the levels measured near the banana plantations in Chile.

Trifluthrin was measured in the state network with detectable levels measured in 3.4–11.1% of samples from 2018 to 2022, although less than 1.5% of those samples had quantifiable levels [53–57]. Trifluthrin was measured in one sampling campaign in 1997 in the foothills above the San Joaquin Valley, with concentrations ranging from 0.03 to 0.064 ng/m^3^ [66]. This compound was also measured at a Canadian Artic Station at very low concentrations, with a mean concentration of 0.043 ± 0.056 pg/m^3^ [67].

The California monitoring network also measured for several additional compounds from 2018 to 2022 we included in the list we measured for that were not detected, all with detection rates typically less than 2% of the state collected samples, with no samples having quantifiable levels, specifically permethrin (0.2–1.6%), dimethoate (0–1.6%), oxyfluorfen (0.2–5.4%), and (S)-metolachlor (0–1.6%) [53–57]. Given that low percentages of samples detected, all at levels below quantification, it is not surprising that these compounds were not detected in our samples. Additionally, the state monitoring program uses larger pumps able to pull a greater volume of air through the samplers. The use of personal samples limits the amount of air that can be collected, and thus stationary sampling campaigns using high-volume samplers are often able to detect levels below 1 ng/m^3^, whereas our limits of detection were much higher than this.

To date, we have not located studies measuring air concentrations of Burprofezin, or Penthiopyrad for comparison. We note that all of these samples were collected in the general season of pesticide applications, but we had no way of knowing if any pesticides were applied in the communities included on the sampling days.

### Potential for adverse health effects

Varying amounts of information are available regarding the potential for adverse health effects from the compounds measured in the study. Chlorpyrifos is clearly demonstrated to have a number of adverse health effects, and as a result, tolerance levels on food products have been revoked. The most recent EPA risk assessments considers both AChE inhibition and potential for neurodevelopmental effects as the primary endpoints of concern [68].

Another compound with considerable evidence for adverse human health impacts is 1,3-dichloropropene, a halogenated organic compound listed as a carcinogen under Proposition 65 and classified as a likely human carcinogen by the US EPA [69]. There are active efforts to reduce use in California [70].

While the toxicity of the other compounds has been less studied, there is the potential for adverse health impacts. Buprofezin, a thiadiazine insecticide that acts on immature insect developmental stages by interfering with cuticle production [71], has been found to cause malformations in embryos of catfish larvae [72]. The compound has also been tested in mice and human cell lines. The study conducted in mice found accumulation of the compound in the liver, as well as increased levels of methane dicarboxylic aldehyde, an indicator of an oxidative stress response in the liver [73]. The cell-line tests indicated reactive oxygen species (ROS) generation and attenuation of adenosine triphosphate (ATP). Altered energy production in the liver is associated with numerous metabolic diseases [74]. Liver toxicity has also been noted as a concern by the World Health Organization [75].

Developmental toxicity has been observed in both rats and mice for the fungicide pyrimethanil [76]. Also of concern is the potential for endocrine disruption. A study in zebrafish [77] found altered expression of genes related to the endocrine system, as well as oxidative stress. In treefrogs [78], a range of toxic effects was observed, including interferences with normal sexual differentiation, indicating potential endocrine disruption. Endocrine disruption was also evaluated through high-throughput toxicity testing, specifically finding AhR-agonist activity [79] and antiandrogen and androgen effects [80].

Of the detected compounds, Penthiopyrad, a succinate dehydrogenease inhibitor fungicide, appears to be the least studied. One study found developmental concerns in zebrafish, including deformities during development and poorer swimming, as well as showing changes in lipid metabolism [81]. A second zebrafish study also found differences in lipid metabolism [82], as well as oxidative stress in the liver and expression of mitochondrial respiratory complexes. This compound should be further studied to determine if similar findings are observed in a mouse or other mammal models.

## Conclusions

This study shows that individuals in agricultural regions of the San Joaquin Valley of California continue to be exposed to pesticides. This study suggests that we need to expand which pesticides we are measuring since personal exposure was observed for compounds not regularly measured in routine monitoring programs. Additionally, these compounds should undergo additional toxicity testing as the existing literature does show some signs of toxicity. The California Department of Pesticide Regulation has implemented policies to reduce pesticide exposures [83] and to promote Integrated Pest Management, a method that supports reduced pesticide use through alternative pest management strategies [84].

## Data Availability

Data is available from the corresponding author on reasonable request.
